# A study of professional practices, attitudes and barriers to blended tube feeding in Australia and New Zealand

**DOI:** 10.1111/1747-0080.12909

**Published:** 2024-10-21

**Authors:** Claire Reilly, Nicole Ross, Stacey Watene, Rachel Lindeback, Tanya Coelho, Usha Krishnan, William Pinzon Perez, Neha Chandrasekar, Jason Yap, Lina Breik, Fiona Arrowsmith

**Affiliations:** ^1^ School of Health & Rehabilitation Sciences The University of Queensland Brisbane Queensland Australia; ^2^ Department of Dietetics and Food Services Queensland Children's Hospital Brisbane Queensland Australia; ^3^ Nutrition and Food Services Gold Coast Hospital and Health Service Gold Coast Queensland Australia; ^4^ St George Hospital Kogarah New South Wales Australia; ^5^ Perth Children's Hospital Perth Western Australia Australia; ^6^ School of Clinical Medicine, Discipline of Paediatrics, Faculty of Medicine and Health University of New South Wales Sydney New South Wales Australia; ^7^ Department of Paediatric Gastroenterology Sydney Children's Hospital Sydney New South Wales Australia; ^8^ QCIF Bioinformatics, Institute for Molecular Bioscience The University of Queensland Brisbane Queensland Australia; ^9^ Faculty of Medicine and Health The University of New South Wales Sydney New South Wales Australia; ^10^ Concord Repatriation General Hospital Sydney New South Wales Australia; ^11^ Children's Intestinal Rehabilitation & Clinical Nutrition The Royal Children's Hospital Melbourne Victoria Australia; ^12^ University of Melbourne Melbourne Victoria Australia; ^13^ Home Enteral Nutrition Care Melbourne Victoria Australia; ^14^ Dietitian for Kids Sydney New South Wales Australia

**Keywords:** blended tube feeding, enteral nutrition, health personnel, health survey, scope of practice

## Abstract

**Aims:**

This study investigates the utilisation of blended tube feeding by health professionals in Australia and New Zealand, assessing factors influencing its implementation following the Australasian Society of Parenteral and Enteral Nutrition blended tube feeding consensus statement.

**Methods:**

A cross‐sectional survey was conducted targeting health professionals across Australia and New Zealand. The survey comprised 35‐questions including multiple choice, Likert scales and open‐ended responses, to gain insights into blended tube feeding practices and perspectives. The effect of the health professional factors on outcomes was explored in pairs with a series of Chi‐squared tests. Odds ratios (ORs) were calculated using standard univariate logistic regression. An exploratory content analysis was used to code the open‐ended text responses to the survey questions which were then categorised and further synthesised into overarching themes.

**Results:**

Out of 89 health professionals who completed the survey, the majority were dietitians, 63% reported managing fewer than five patients using blended tube feeding within their services. Parental request was the leading reason for adoption. Notable barriers included clinician time constraints, resource limitations and a lack of formal guidelines. Some health professionals considered the primary risk associated with blended tube feeding to be poor growth and/or weight loss. Professional development was pivotal in increasing confidence and advocating for blended tube feeding, with significant correlations observed between blended tube feeding training and clinical practice.

**Conclusions:**

This study emphasises the essential role of education, resource availability and institutional policy in promoting blended tube feeding practices for health professionals. Findings suggest that focusing on professional development and standardised resources could significantly enhance knowledge, confidence and competence of health professionals in blended tube feeding application. The outcomes point towards the need for a coordinated approach to support evidence‐based blended tube feeding practices, aligning with the Australasian Society of Parenteral and Enteral Nutrition blended tube feeding resources and recommendations.

## 1 INTRODUCTION

Blended tube feeding is increasingly recognised as an alternative to commercial enteral formulas for its potential to enhance quality of life among adult and paediatric patients worldwide.^1^, ^2^, ^3^, ^4^, ^5^ A preference for ‘real food’ alternatives; and encouraging evidence on gastrointestinal health and microbiome benefits are contributing to its rising adoption.^1^, ^6^, ^7^, ^8^ Initial concerns regarding microbial contamination, tube blockages and nutritional adequacy are being reconsidered in recent research, driving a growing endorsement for blended tube feeds under health professional supervision.^1^, ^7^, ^9^, ^10^, ^11^, ^12^, ^13^

In recent years, international research and evolving clinical practice have increasingly recognised the importance of alternative nutrition support options, leading to significant changes in the acceptance and implementation of blended tube feeding. Leading international health professional organisations have published position papers, practice recommendations, guidelines and toolkits supporting the adoption of blended tube feeding as a viable nutrition support option for both children and adults.^1^, ^14^, ^15^, ^16^, ^17^ The viscosity of blended tube feeds is now categorised using the internationally accepted International Dysphagia Diet Standardisation Initiative (IDDSI) standards, further contributing to the broader acceptance of blended tube feeding on an international level.

While international research has explored health professional attitudes toward blended tube feeding,^18^, ^19^, ^20^, ^21^, ^22^, ^23^ the Australia and New Zealand context remains underrepresented. A cross‐sectional study in Australia showed that 40% of health services have incorporated blended tube feeds into patient care, despite limited data on blended tube feeding practices in both Australia and New Zealand.^24^ Following the publication of Australasian Society of Parenteral and Enteral Nutrition blended tube feeding consensus statement,^25^ a landmark effort to standardise care in Australia and New Zealand, there is an urgency to investigate current health professionals' perceptions and practices in blended tube feeding.

This study aims to assess blended tube feeding utilisation by health professionals in Australia and New Zealand and factors influencing its use.

## 2 METHODS

Ethics approval for this study was obtained from the Children's Health Queensland Hospital and Health Service Human Research Ethics Committee (EX/21/QCHQ/80828). This study was conducted as a cross‐sectional survey targeting health professionals in paediatric and adult care within Australia and New Zealand. This study is reported and informed by the Checklist for Reporting Of Survey Studies (CROSS) (S1).^26^

**FIGURE 1:**
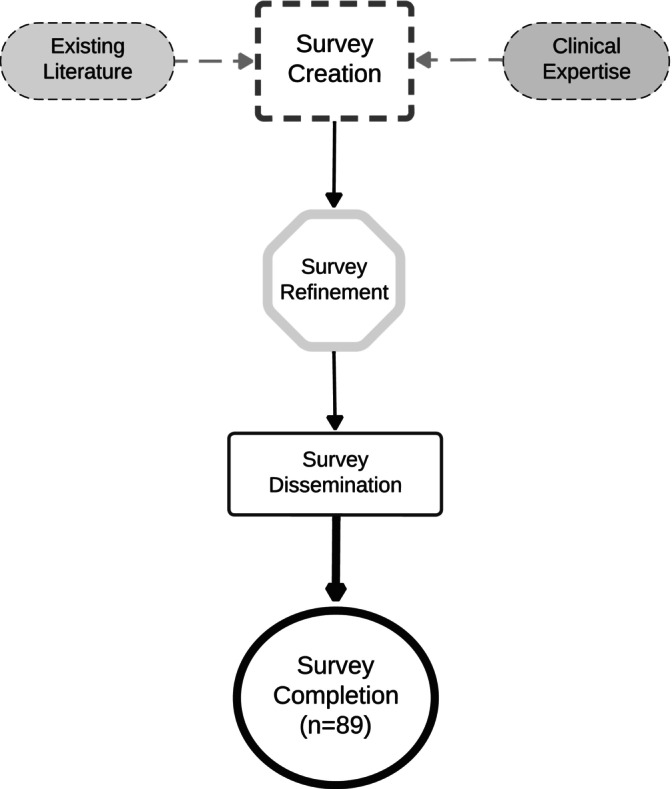
Flowchart of the survey creation.

The survey, consisting of 35 questions (multiple choice, Likert scales and open‐ended), was collaboratively developed by a multidisciplinary team from the Australasian Society of Parenteral and Enteral Nutrition blended tube feeding sub‐committee, drawing upon combined clinical expertise and the existing blended tube feeding literature, as described in Figure 1. The decision was made to omit distinctions between whole food and commercial blended tube feeds in the survey questions to obtain a broad understanding of blended tube feeding practices. The survey was hosted online via Google Forms, with the estimated completion time being 10–15 min, and was distributed via email through extensive professional networks including Australasian Society of Parenteral and Enteral Nutrition members, Australian Society for Paediatric Gastroenterology Hepatology and Nutrition members, Gastroenterological Society of Australia members, Queensland Health Paediatric Special Interest Group, National Disability Insurance Scheme Private Health Professionals and the Queensland Health Enteral Nutrition Community of Practice members. Participation was voluntary, no incentives were offered, and consent was implied upon survey completion. The survey, detailed in the Supporting Information, was disseminated via email in July 2022 for a period of two months. Data were collected anonymously and reported collectively, no identifiable personal data was included.

Summary statistics were presented of all the relevant variables. The effect of the health professional factors on outcomes was explored in pairs with a series of Chi‐squared tests due to the categorical nature of the variables. Odds ratios (ORs) were calculated using standard univariate logistic regression using the R function ‘glm’ as exp(*β*), *β* being the estimate for the logistic regression model. All data analyses were performed in R version 4.3.3 (29 February 2024 UCRT) with RStudio Desktop version 2023.6.0.421. Statistical significance was reported at 5%.

An exploratory content analysis approach was utilised to analyse the open‐ended responses to the survey questions, guided by the principles of Graneheim and Lundman.^27^ Meaningful units were identified, condensed while preserving their core content, and then coded. These codes were then grouped into categories based on similarities and synthesised into overarching themes. The data were organised and managed using Microsoft Excel (Microsoft Corporation, version 2024), which allowed for the creation of a structured matrix to systematically categorise and cross‐reference codes and themes, facilitating the visualisation of emerging patterns.

Three independent authors in the team conducted an initial detailed review of the responses, each independently identifying and extracting core themes and patterns. These themes were further distilled and categorised collaboratively using Microsoft Excel, where the three authors systematically refined the themes by reviewing and re‐evaluating the codes, grouping similar concepts and organised them into hierarchical categories. This process involved iterative discussions and consensus‐building meetings to ensure the categorisation accurately reflected the data and captured the underlying patterns. Rigorous coding measures, including cross‐checking, ensured the accuracy and validity of the findings.

## 3 RESULTS

Table 1 presents the characteristics of the 89 health professionals that completed the survey, with the majority (84.1%) being dietitians, followed by paediatric gastroenterologists (12.3%), speech pathologists (2.2%) and general practitioners (1.1%). Table 2 presents the use of blended tube feeding within a health service. Most services using blended tube feeds were less extensive, with 63% having fewer than five patients per service.

**TABLE 1:** Survey respondent characteristics.

	% (*n*)
Geographic location (*n* = 89)	
Australia	82 (73)
New Zealand	18 (16)
Area of practice (*n* = 89)	
Paediatrics	53 (47)
Adults	22 (20)
Mixed (paediatrics & adults)	25 (22)
Clinical service^a^ (*n*)	
Tertiary hospital	55 (49)
Regional hospital	19 (17)
Rural hospital	2 (2)
Community	17 (15)
Private practice	15 (13)
Disability	1 (1)
Years of clinical experience (*n* = 88)	
0–4 years	11 (10)
5–10 years	34 (30)
11–20 years	30 (26)
>20 years	25 (22)
Experience level with BTF (*n* = 89)	
Minimal	54 (48)
Intermediate	26 (2)
Advanced	20 (18)
Knowledge of BTF (*n* = 89)	
Strongly agree	10 (9)
Agree	37 (33)
Neither agree nor disagree	27 (24)
Disagree	19 (17)
Strongly disagree	7 (6)
Confidence in the assessment and provision of BTF (*n* = 88)	
Strongly agree	12 (11)
Agree	33 (29)
Neither agree nor disagree	24 (21)
Disagree	25 (22)
Strongly disagree	6 (5)
Confidence discussing BTF with medical teams (*n* = 89)	
Strongly agree	20 (18)
Agree	47 (42)
Neither agree nor disagree	15 (13)
Disagree	12 (11)
Strongly disagree	6 (5)
Professional development received in BTF (*n* = 85)	
Yes	67 (57)
No	33 (28)

a The total number of types of clinical services chosen is 97, this is due to some of the 89 respondents choosing more than one clinical service in the survey.

**TABLE 2:** Results on the use of blended tube feeding within a service.

	% (*n*)
Does your service/organisation/practice support the use of BTF with your patients/clients? (*n* = 88)	
No	28 (25)
Yes for both inpatients and outpatients	15 (13)
Yes for outpatients only	57 (50)
Yes for inpatients only	0
Does your service have a documented policy and procedure for clinicians who support BTF? (*n* = 87)	
Yes	16 (14)
No	71 (62)
Unsure	13 (11)
Do you currently access the AuSPEN BTF resources for clients/patients and health professionals? (*n* = 89)	
Yes	46 (41)
No	54 (48)
Does your service use standardised BTF patient resources for patients/carers/families? (*n* = 84)	
Yes	45 (38)
No	55 (46)
Does your medical/specialist team recommend BTF for clients/patients as a treatment option? (*n* = 87)	
Yes	29 (25)
No	71 (62)
Do you provide consumables to your patients/clients on BTF? (*n* = 75)	
Yes	44 (33)
No	56 (42)

Abbreviations: AuSPEN, Australasian Society of Parenteral and Enteral Nutrition; BTF, blended tube feeding.

Blended tube feeding was initiated upon parental request (73%), for managing gastrointestinal symptoms (54.1%), such as reflux (54.1%) or intolerance to commercial enteral formula (51.4%). Constraints to blended tube feeding use included limited patient knowledge of food safety and hygiene (76.1%), limited access to the required equipment (e.g., blender) (81.8%), jejunal feeds (75%) and small tube size (<14 French) (63.6%). Despite this, the majority of respondents (63%) reported positive outcomes for their patients using blended tube feeds including observations of decreased vomiting (73.3%), constipation (73.3%) and retching (51.7%). Some respondents (27%) reported weight loss or poor growth as a negative change related to blended tube feeding. These respondents were predominantly experienced paediatric dietitians from tertiary hospital settings (54%), although the majority of these health professionals (71%) still endorsed blended tube feeding benefits.

The most common cited barriers were health professional time commitment (63.2%), limited resources (45.6%), lack of practical guidelines (52.9%) and a need for further education/training (50%). A vast majority (93.2%) would welcome Australian and New Zealand blended tube feeding guidelines to enhance confidence. Professional development was largely self‐initiated (60%), with webinars (52.3%) and peer learning (46.2%) being the preferred modalities. Respondents favoured webinars as their preferred method for receiving blended tube feeding professional development (78.9%).

**FIGURE 2:**
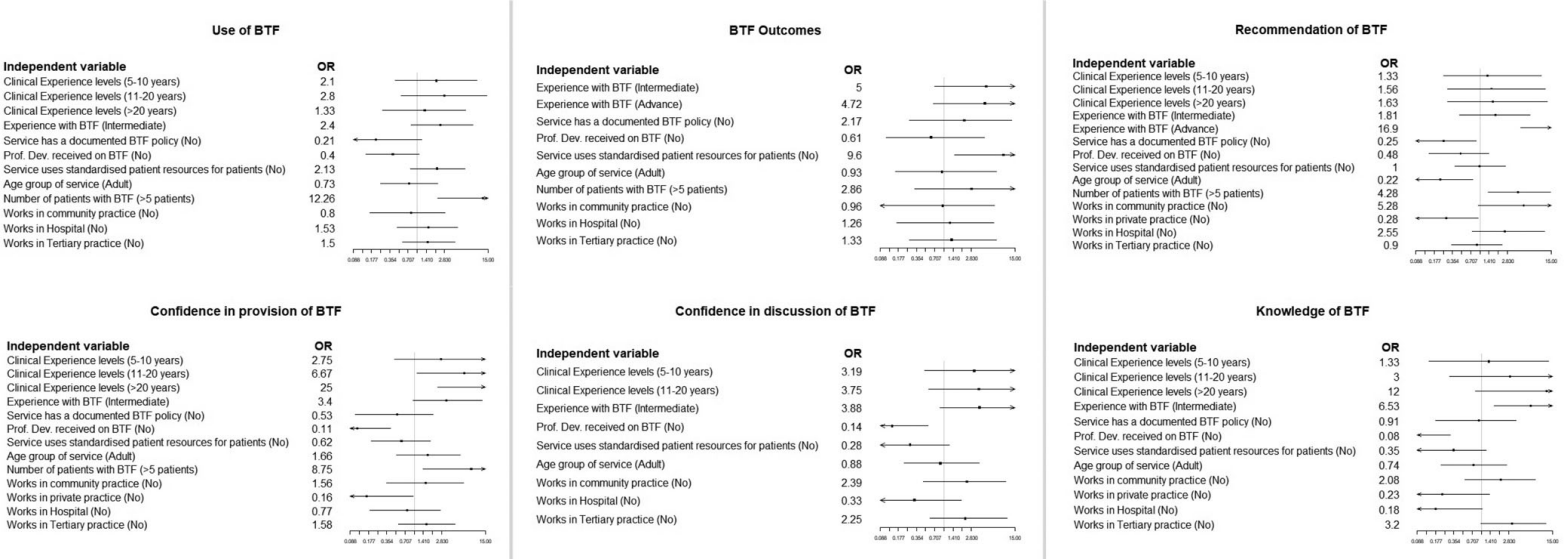
The odds ratios and corresponding 95% confidence intervals for the associations between the use of blended tube feeding, blended tube feeding confidence, knowledge of blended tube feeding, outcomes and recommendation of blended tube feeding. BTF, blended tube feeding.

Figure 2 displays the ORs and corresponding 95% confidence intervals (CIs) for the associations between the use of blended tube feeding, blended tube feeding confidence, knowledge of blended tube feeding, outcomes and recommending blended tube feeding. The statistical analysis revealed significant associations within the themes of blended tube feeding usage, confidence, knowledge and recommendation practices among health professionals.

In services where more than five patients were managed with blended tube feeds, health professionals demonstrated a significantly higher likelihood of blended tube feeding utilisation than services with five patients or less (OR = 12.2, 95% CI [2.177, 231.4], *p* = 0.02). Notably, there was a substantially larger likelihood in exhibiting confidence among health professionals with over two decades of experience (OR = 25, 95% CI [2.44, 661.82], *p* = 0.016) and professionals with 11 to 20 years of experience (OR = 6.67, 95% CI [1.12, 56.43], *p* = 0.049) as opposed to those with less than 5 years of experience. This underscores a clear link between clinicians' experience with blended tube feeding and their willingness to engage in blended tube feeding practices.

A pivotal component for supporting confidence in blended tube feeding was identified as continuous professional development. Health professionals who participated in blended tube feeding‐specific training and education were more likely to exhibit confidence not only in the use of blended tube feeds but also in discussing its implementation with their medical teams than those that did not participate in such training (OR for confidence in using blended tube feeds = 8.77, 95% CI [2.47, 37.04], *p* < 0.001; OR for confidence in discussion = 7.14, 95% CI [1.84, 31.25], *p* = 0.006).

The results showed that health professionals with an intermediate level of blended tube feeding experience were considerably more likely to be knowledgeable about blended tube feeding than their peers with minimal experience (OR = 6.53, 95% CI [1.62, 34.16], *p* = 0.013). This highlights the effectiveness of experiential learning, which is further supported by the fact that engaging in professional development significantly contributes to blended tube feeding knowledge more than those that did not engage in professional development (OR = 12.5, 95% CI [3.247, 58.82], *p* < 0.001).

In examining the likelihood of recommending blended tube feeding, health professionals with advanced blended tube feeding experience were significantly more inclined to work in teams that advocate for its use (OR = 16.89, 95% CI [4.73, 71.52], *p* < 0.001) than their peers with minimal experience. Moreover, the existence of a formalised blended tube feeding policy within a service boosted the chances of health professionals recommending blended tube feeding (OR = 4.00, 95% CI [1.155, 14.49], *p* = 0.029) when compared to services that did not have a formal blended tube feeding policy.

Particularly in paediatric services or private practice settings, there was a higher tendency to support blended tube feeding use than in adult and public settings, respectively, suggesting that specialised clinical areas and service policies play a crucial role in blended tube feeding endorsement (OR for paediatric services = 4.5, 95% CI [1.29, 21.27], *p* = 0.03; OR for private practice = 3.56, 95% CI [11.06, 12.50], *p* = 0.004).

Content analysis delineated key themes from five open‐ended survey questions as described in Table 3. Provision of consumables (e.g., syringes) for blended tube feeding was common, though few services offered equivalent supplies provided to non‐blended tube feeding patients, with even fewer providing feeding pumps. Feedback on Australasian Society of Parenteral and Enteral Nutrition blended tube feeding resources was predominantly positive; however, there were appeals for more detailed resources, such as recipe inclusion. Noted barriers included a lack of service support, concerns over food hygiene and the application of blended tube feeding in complex medical patients. Specific contraindications for blended tube feeding were identified in immunocompromised patients, those with limited health literacy, and those experiencing food insecurity. Despite these challenges, health professionals reported blended tube feeds enhanced quality of life, underscoring the necessity for patient‐centred care. Several health professionals called for the need for formal endorsement by professional organisations to mitigate barriers and facilitate widespread blended tube feeding use.

**TABLE 3:** Content analysis of open‐ended questions.

Theme	Category	Illustrative quotes
Effectiveness of the Australasian Society of Parenteral and Enteral Nutrition BTF resources	Beneficial	*They are very useful to support establishment and monitoring of BTF [P84]*
	Inadequate	*Quite generic and not enough detail on recipe planning and meeting requirements [P47]*
Endorsement of the Australasian Society of Parenteral and Enteral Nutrition BTF resources	Service constraints	*Our service does not recommend it, but if parents decide to try it we will support them to do it as safely as possible [P81]*
	Perceived risk	*I think patients need to be aware of risks including poor growth and should be counselled [P6]*
Contraindications for use of BTF	Safety	*Overcrowded living so unable to practice food hygiene [P65]*
		*Immunocompromised cohort (e.g. oncology), NGTs [nasogastric tubes] [P77]*
		*Food insecurity, health illiteracy [P38]*
Use of BTF	Quality of life	*Improves parent child relationship – sense of being able to feed their child. The person using the blend can develop a better relationship with foods [P71]*
	Time intensive	*The additional time spent planning a regime saves time as the children are less unwell and require less input long term often [P41]*
	Commitment	*I think if it is done well it requires a lot of time and a very health literate, motivated parent [P5]*

Abbreviation: BTF, blended tube feeding.

## 4 DISCUSSION

This study provides a comprehensive insight into the practices and attitudes of health professionals towards the use of blended tube feeding in Australia and New Zealand. The results reveal that professional development, experience and institutional policy significantly influence the adoption of blended tube feeding. Health professionals with extensive blended tube feeding experience and those working within a supportive policy framework showed a greater predisposition to integrate blended tube feeding into patient care. The findings resonate with international research,^19^ indicating a supportive trend for blended tube feeds use in clinical settings, with many health professionals reporting support for blended tube feeding within their services. The presence of blended tube feeding policies, however, is not ubiquitous, which may account for the observed variability in blended tube feeding application among health professionals with less blended tube feeding experience. A notable number of respondents had a service with fewer than five patients on blended tube feeds, pointing toward scope for wider application.

Consistently, the health professionals surveyed recognised patient benefits from blended tube feeding usage, paralleling findings from similar studies.^18^, ^19^, ^20^ Identified hurdles included resource scarcity, insufficient training, and the absence of definitive guidelines, all of which echo the global challenges faced in the implementation of blended tube feeding.^18^, ^19^, ^21^, ^22^, ^28^ The identification of weight loss or poor growth was identified as a potential risk of blended tube feeding, despite evidence suggesting its efficacy in improving patient outcomes.^7^, ^29^ This risk was noted primarily by experienced paediatric dietitians, underscoring the importance of close monitoring when used in medically complex patients in tertiary care settings.

Professional confidence and knowledge emerged as core themes in the adoption of blended tube feeding, similar to other research.^19^, ^21^ Health professionals demonstrate a keen interest in expanding their understanding of blended tube feeding,^30^ recognising that enhanced knowledge in this area is crucial for optimal patient care.^31^

Respondents primarily acquired their blended tube feeding knowledge through self‐directed and peer‐assisted learning, similar to other research.^19^, ^21^ However, the nature of these self‐teaching methods remains unspecified, especially since many respondents were not familiar with the Australasian Society of Parenteral and Enteral Nutrition blended tube feeding resources or related professional guidelines. This gap reflects broader concerns within the health profession regarding insufficient training and confidence in blended tube feeding practices, aligning with previous research.^7^, ^21^

The need for structured education is paramount to further advance the uptake of blended tube feeding, such as comprehensive training to screen suitable patients for blended tube feeding,^28^ while the lack of robust evidence continues to impede its broader uptake.^18^, ^21^, ^22^

The prevailing enthusiasm for blended tube feeding is mirrored in the patient community, with parental advocacy being a primary driver for its consideration in line with previous research.^20^, ^21^, ^28^, ^30^ The positive outcomes reported by patients using blended tube feeds was seen in several research studies.^5^, ^13^, ^20^, ^29^, ^32^, ^33^, ^34^ The absence of support, guidance and knowledge from health professionals may lead patients to seek information from potentially unreliable sources such as social media^32^ posing potential risks and misinformation. This is of particular relevance to paediatric health professionals, as blended tube feeding adoption among this demographic appears significant and is likely to grow, emphasised by a recent study revealing that 90% of paediatric patients on gastrostomy tube feeds were using blended tube feeds.^31^

In terms of the qualitative analysis, it corroborates the survey data by providing nuanced insights into the experiences and challenges health professionals encounter with blended tube feeding. The respondents had a cautious stance, when compared to international counterparts,^1^ highlighting the need for greater clinical evidence and formal Australia and New Zealand guidelines on clinical practice. Interestingly, the respondents' confidence levels and reasons for using blended tube feeds align with those reported in other published surveys of health professionals.^18^, ^23^ An overwhelming number of the respondents agreed that they would change their perception of blended tube feeding if there was more evidence or guidelines available.

While this study makes valuable insights in the current adoption of blended tube feeding in Australia and New Zealand, it is important to recognise its limitations. The survey instrument used in this study was not validated, potentially impacting the reliability of the findings. Additionally, the unknown response rate and the possibility of selection bias limit the generalisability of the results. Furthermore, relying on respondents' self‐reported knowledge, confidence, and experience may have introduced bias into the data. A further limitation is the absence of data on patients' specific health conditions managed by health professionals, which may impact the generalisability of the findings across diverse different health conditions and care complexities. However, the innovative approach taken in the Australia and New Zealand context, coupled with the substantial number of participants, enhances the significance of this study. Furthermore, the inclusion of open‐ended questions provided deeper insights into the perspectives and practices related to blended tube feeding.

In conclusion, this study underscores the pivotal role of education, resource development and evidence‐based policy in supporting blended tube feeding practices for health professionals. This study acts as a call to action for comprehensive professional development and the establishment of standardised resources to enhance the confidence and competence of health professionals in blended tube feeding. With the advent of new guidelines from the European Society for Paediatric Gastroenterology, Hepatology and Nutrition^14^ and the American Society for Parenteral and Enteral Nutrition,^1^ an opportunity arises to assess shifts in practice and perception, potentially leading to a more unified and evidence‐informed approach to blended tube feeding.

## AUTHOR CONTRIBUTIONS

**CR**: Primary conceptualisation, methodology, data curation, led qualitative analysis, drafted and revised all versions of the manuscript. **NR**: Primary conceptualisation, recruitment, methodology, data curation, investigation, contributed to qualitative analysis, reviewed and revised all versions of the manuscript. **SW**: Primary conceptualisation, recruitment, data curation, contributed to qualitative analysis, reviewed a draft of the final manuscript. **RL**: Primary conceptualisation, recruitment, methodology, reviewed a draft of the final manuscript. **NC**: Conceptualisation, recruitment. **TC**: Primary conceptualisation, recruitment, methodology. **JY**: Contributed to data analysis, recruitment, reviewed a draft of the final manuscript. **UK**: Methodology, recruitment, reviewed two drafts of the final manuscript. **LB**: reviewed the final two drafts of the final manuscript. **WPP**: Conducted all statistical analyses, reviewed the final two drafts of the manuscript. **FA**: Primary conceptualisation, methodology, recruitment, data curation, contributed to qualitative analysis, reviewed all drafts of the manuscript and provided substantial contributions to revising the manuscript. All authors are in agreement with this manuscript and declare that the content has not been published elsewhere.

## CONFLICT OF INTEREST STATEMENT

Ms Rachel Lindeback: speaker for Cardinal Health (two talks on blended tube feeding), sponsorship from Vitaflo to attend the International Renal Conference in Manchester, previous speaker for Biogen, Roche and SMA Australia, however, none related to this project. Ms Lina Breik: has undertaken consultative and educational work with Abbott Australia, Nutricia Australia and Fresenius Kabi Australia, however, none related to this project. The other authors declare no conflicts of interest.

## ETHICS STATEMENT

Ethics approval was obtained from the Children's Health Queensland Hospital and Health Service, Human Research Ethics Committee (Reference number EX/2021/QCHQ/80828).

## Supporting information


**Data S1:** Supporting Information.

## Data Availability

The data that support the findings of this study are available from the corresponding author upon reasonable request.
