# Supplementary material for: A study of professional practices, attitudes and barriers to blended tube feeding in Australia and New Zealand
Source: Nutr Diet. 2024 Oct 21;82(2):143–51. doi: 10.1111/1747-0080.12909 (PMC11973618; doi:10.1111/1747-0080.12909)
Supplement: Supplementary file 1 — Data S1: Supporting Information. [file NDI-82-143-s001.pdf]

Blended Tube Feeds - Perceptions and Practice - Australia and New Zealand

We aim to collect current perceptions and practices in relation to the use of blended tube feeds across Australian and New Zealand Health professionals

1. Email

____

2. Please choose a profession which best suits you

Tick all that apply.

- [ ] Dietitian
- [ ] General Practitioner
- [ ] Paediatrician
- [ ] Paediatric Gastroenterologist
- [ ] Adult Gastroenterologist
- [ ] Speech Pathologist
- [ ] Nurse
- [ ] Other: _______________________________

3. Do you practice in Australia or New Zealand?

Mark only one oval.

- ( ) Australia
- ( ) New Zealand
- ( ) Other: _______________________________

4. Do you work for a Paediatric, Adult or Mixed (Paediatric/Adult) Service? *

Tick all that apply.

- [ ] Paediatric
- [ ] Adult
- [ ] Mixed

5. What is the name of the organisation you work in?

____

6. What type of service do you work in?

Tick all that apply.

- [ ] Tertiary Hospital
- [ ] Secondary/Regional Hospital
- [ ] Rural Hospital
- [ ] Community
- [ ] Private Practice
- [ ] General Practice
- [ ] Other: ______________________________

7. How many years clinical experience do you have?

Tick all that apply.

- [ ] 0-4 years
- [ ] 5-10 years
- [ ] 11-20 years
- [ ] >20 years

8. How many enterally fed clients/patients does your service manage?

____

9. Does your service/organisation/practice support the use of blended tube feeds (BTF) with your clients/patients ?

Tick all that apply.

- [ ] No
- [ ] Yes for both inpatients and outpatients
- [ ] Yes for outpatients only
- [ ] Yes for inpatients only

10. Please estimate the number of clients/patients in your service that are tube fed and utilising BTF as a part of their nutrition regimen (this can be full BTF or using BTF and commercial formulas together as a part of nutritional regime)

____

11. What is your experience level with BTF

Tick all that apply.

- [ ] Low, minimal involvement with commencing or managing patients on BTF
- [ ] Intermediate, use in clinical practice with support/supervision
- [ ] Advanced, significant involvement in commencing and managing patients on BTF

12. If you have not used BTF before, are you interested in doing so?

Tick all that apply.

- [ ] Yes
- [ ] No

13. I am knowledgeable in BTF

Tick all that apply.

- [ ] Strongly Agree
- [ ] Agree
- [ ] Neither Agree nor Disagree
- [ ] Disagree
- [ ] Strongly Disagree

14. I am confident in the assessment and provision of nutritionally adequate BTF

Tick all that apply.

- [ ] Strongly Agree
- [ ] Agree
- [ ] Neither Agree nor Disagree
- [ ] Disagree
- [ ] Strongly Disagree

15. I am confident to discuss the use of BTF with medical staff and specialists

Tick all that apply.

- [ ] Strongly Agree
- [ ] Agree
- [ ] Neither Agree nor Disagree
- [ ] Disagree
- [ ] Strongly Disagree

16. I would feel more confident in the provision of BTF if there was an Aust/NZ guideline and standardised resources

Tick all that apply.

- [ ] Strongly Agree
- [ ] Agree
- [ ] Neither Agree nor Disagree
- [ ] Disagree
- [ ] Strongly Disagree

17. Have you received any professional development around the provision of BTF?

Tick all that apply.

- [ ] Yes
- [ ] No

18. If Yes, please indicate below the type of professional development you have participated in

Tick all that apply.

- [ ] Self-directed learning
- [ ] Peer learning
- [ ] Learning from patients/families
- [ ] Workplace education sessions
- [ ] Journal articles
- [ ] COnferences
- [ ] On-line Webinars
- [ ] Other: ______________________

19. If No, and you are interested in professional development on the use of BTF in patients, what would be your preferred learning method?

Tick all that apply.

- [ ] Printed resources
- [ ] Webinar
- [ ] Workshop
- [ ] Resource packs

20. Do you currently access the AuSPEN BTF resources for clients/patients and health professionals?

Mark only one oval.

- ( ) Yes
- ( ) No

21. If you answered yes to the above questions, do you have any comments on the AuSPEN BTF resources?

____
____
____
____
____

22. Does your service have a documented policy and procedures for clinicians who support BTF?

Tick all that apply.

- [ ] Yes
- [ ] No
- [ ] Unsure

23. If no, what are the barriers (perceived or real) to supporting BTF in your service (if any)?

Tick all that apply.

- [ ] Limited resources
- [ ] Clinician time commitment
- [ ] Limited education/training
- [ ] Not supported in the workplace
- [ ] Limited evidence
- [ ] Lack of practical guidelines for safe practice
- [ ] I do not support the use of BTF in clinical practice
- [ ] Not supported by Dietitian's Australia or Dietitian's Association of New Zealand
- [ ] Other: ______________________

24. If Yes, what sort of documents does your service use?

Tick all that apply.

- [ ] BTF use consent forms
- [ ] Local BTF guidelines
- [ ] British Dietetic Association BTF guidelines
- [ ] Other: ______________________

25. Does your service use standardised patient resources for patients/carers/families?

Mark only one oval.

- ( ) Yes
- ( ) No

26. If Yes, what resources are routinely used?

____

27. In what clients/patients population do you use blended tube feeds?

____

28. Does your medical/specialist team recommend BTF for clients/patients as a treatment option?

Mark only one oval.

- ( ) Yes
- ( ) No

29. If yes, are there common reasons for this?

Tick all that apply.

- [ ] Reflux
- [ ] Commercial formula intolerance
- [ ] Parental request
- [ ] Dieteary restriction or preference
- [ ] Gastrointestinal symptom management
- [ ] Not applicable
- [ ] Other: ________________________________

30. What do you see as contraindications for use of BTF with clients/patients?

Tick all that apply.

- [ ] Poor growth
- [ ] Medical team approval
- [ ] Continuous feeds
- [ ] Jejunal feeds
- [ ] Small size (<14 french) gastrostomy
- [ ] Newly inserted gastrostomy tract
- [ ] Fluid restrictions
- [ ] Patient has limited knowledge about food safety and hygiene
- [ ] Patient/carer has limited access to the required equipment (e.g. blender)
- [ ] Too time consuming for patient/carer
- [ ] Other: ____________________

31. Do you provide consumables to your clients/patients on BTF?

Mark only one oval.

- ( ) Yes
- ( ) No

32. If Yes, what consumables do you provide?

____

33. What is the general outcome of using BTF in your clients/patients with gastrostomy feeds

Tick all that apply.

- [ ] Positive
- [ ] Negative
- [ ] Not applicable, I dont use BTF in my patients

34. Have your clients/patients on BTF experienced any positive changes or improvement in the following gastrointestinal symptoms since commencing BTF?

Tick all that apply.

- [ ] Retching
- [ ] Vomiting
- [ ] Nausea
- [ ] Bloating
- [ ] Constipation
- [ ] Diarrhoea
- [ ] Abdominal Pain
- [ ] Weight gain/improved growth

35. Have your clients/patients on BTF experienced any negative changes or decline in the following gastrointestinal symptoms since commencing BTF?

Tick all that apply.

- [ ] Retching
- [ ] Vomiting
- [ ] Nausea
- [ ] Bloating
- [ ] Constipation
- [ ] Diarrhoea
- [ ] Abdominal Pain
- [ ] Weight loss/poor growth
- [ ] Other

36. Is there anything else you would like to add about BTF?

Google
